# Docking and Selectivity Studies of Covalently Bound Janus Kinase 3 Inhibitors

**DOI:** 10.3390/ijms24076023

**Published:** 2023-03-23

**Authors:** Haizhen A. Zhong, Suliman Almahmoud

**Affiliations:** 1 Department of Chemistry, The University of Nebraska at Omaha, 6001 Dodge Street, Omaha, NE 68182, USA; 2 Department of Medicinal Chemistry and Pharmacognosy, College of Pharmacy, Qassim University, Buraidah 51542, Saudi Arabia

**Keywords:** Covalent Dock, JAK, binding affinity, anticancer, selectivity

## Abstract

The Janus kinases (JAKs) are a family of non-receptor cytosolic protein kinases critical for immune signaling. Many covalently bound ligands of JAK3 inhibitors have been reported. To help design selective JAK inhibitors, in this paper, we used five model proteins to study the subtype selectivity of and the mutational effects on inhibitor binding. We also compared the Covalent Dock programs from the Schrodinger software suite and the MOE software suite to determine which method to use for the drug design of covalent inhibitors. Our results showed that the docking affinity from 4Z16 (JAK3 wild-type model), 4E4N (JAK1), 4D1S (JAK2), and 7UYT (TYK2) from the Schrödinger software suite agreed well with the experimentally derived binding free energies with small predicted mean errors. However, the data from the mutant 5TTV model using the Schrödinger software suite yielded relatively large mean errors, whereas the MOE Covalent Dock program gave small mean errors in both the wild-type and mutant models for our model proteins. The docking data revealed that Leu905 of JAK3 and the hydrophobic residue at the same position in different subtypes (Leu959 of JAK1, Leu932 of JAK2, and Val981 of TYK2) is important for ligand binding to the JAK proteins. Arg911 and Asp912 of JAK3, Asp939 of JAK2, and Asp988 of TYK2 can be used for selective binding over JAK1, which contains Lys965 and Glu966 at the respective positions. Asp1021, Asp1039, and Asp1042 can be utilized for JAK1-selective ligand design, whereas Arg901 and Val981 may help guide TYK2-selective molecule design.

## 1. Introduction

**Figure 1:**
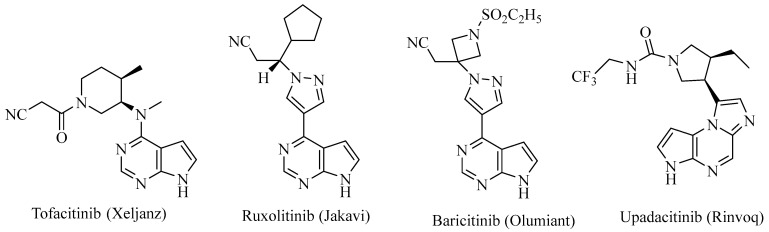
Chemical structures of four US FDA approved JAK inhibitors with brand names in parenthesis.

The Janus kinases (JAKs) are a family of non-receptor cytosolic protein kinases critical for immune signaling [1]. There are four JAK proteins: JAK1, JAK2, JAK3, and tyrosine kinase 2 (TYK2). When extracellular signals in the forms of cytokines, such as interleukins IL-2, IL-4, IL-5, IL-7, IL-15, and IL-21, bind to the cytokine receptors, they cause the dimerization of cytokine receptors. In the dimer, the β chain of the cytokine receptor binds to JAK1 and the gamma chain (γ_c_) cytokine receptor binds to JAK3. The binding causes conformational changes and allows the phosphorylation of JAK1 and JAK3, which in turn phosphorylates the downstream effector signal transducers and activators of the transcription (STAT). The phosphorylated STAT proteins are dimerized and translocated to the nucleus where they bind to the genes that promote cytokine production, leading to inflammation [2]. The JAK dimers that bind to cytokine receptors have been found to be JAK1/JAK3, JAK1/JAK2, JAK1/TYK2, JAK2/TYK2, and JAK2/JAK2. Thus, the JAK/STAT signaling pathway is of fundamental importance in regulating immunity and inflammation [3]. The efficacy of JAK inhibitors has been evaluated for the treatment of rheumatoid arthritis (RA) [4,5], and other inflammatory and autoimmune disorders (e.g., ulcerative colitis and psoriasis) [6,7]. For instance, tofacitinib (Figure 1), a JAK1/2/3 inhibitor, was approved by the US FDA in November 2012 for the treatment of RA [4]. Ruxolitinib (Figure 1), a JAK1/2 and TYK2 inhibitor, was approved in 2011 by the US FDA for the treatment of myelofibrosis [8]. Baricitinib (Figure 1) [9], a JAK1/2 inhibitor, was approved by the US FDA in 2019 for the treatment of RAs for adults who have had an inadequate response to one or more TNF antagonist therapies, and in 2022, it was approved by the USFDA for the treatment of COVID-19 in hospitalized adults who required supplemental oxygen [10]. However, the lack of subtype selectivity of the first generation of JAK inhibitors such as tofacitinib and ruxolitinib has led to undesirable side effects such as infection [11,12], anemia [13], and nasopharyngitis [14] in this otherwise promising class of drugs. Upadacitinib (Figure 1), approved by the USFDA in 2019 to treat RAs and psoriatic arthritis [15,16] in adults where methotrexate was not effective, is a second-generation JAK inhibitor that shows the selectivity of JAK1 over that of JAK2, JAK3, and TYK2.

JAK1, JAK2, and TYK2 are highly expressed in most cell types while JAK3 is mostly expressed in hematopoietic cells [17]. JAK3 was targeted for severe combined immunodeficiency (SCID) therapy [18]. It was noticed that SCID is mostly associated with JAK3 mutation. JAK3 regulates the signaling of the gamma chain (γ_c_) cytokine receptor subunit with interleukin IL-2, IL-4, IL-7, IL-9, IL-15, and IL-21. The γ_c_ subunit is known to make a dimer with another cytokine receptor, an α subunit which is controlled by JAK1, and the γ_c_ subunit can make a trimer with an α subunit and a β subunit such as IL-2R and IL-15R, which is also regulated by JAK1 [11,12,13,14]. The main difference between JAK1 and JAK3 is that the inhibition of JAK1 can cause a wide array of side effects. Therefore, selective JAK3 inhibition while sparing JAK1 is of significance in reducing the side effects of JAK1 inhibition.

**Figure 2:**
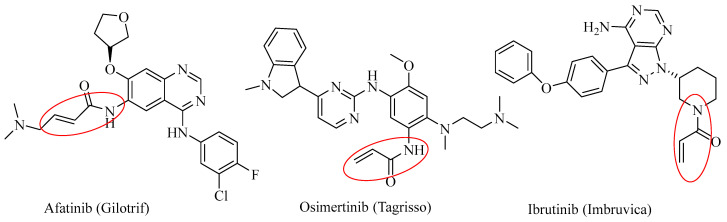
Chemical structures of three US FDA approved covalent drugs with brand names in parenthesis. The Michael addition moiety is highlighted by a red circle.

The traditional strategy for JAK3 inhibitors is to target the ATP-binding region, which was also the binding target of the subtypes JAK1 and JAK2. Due to the highly conserved structural features of the ATP binding pocket, it has been challenging to achieve high selectivity among the JAK family. Many recent developments of JAK3 inhibitors have been focused on a JAK3 unique cysteine residue (CYS909) by forming a covalent bond with JAK3 inhibitors [19,20,21,22,23,24,25,26,27,28,29]. The idea of developing an inhibitor that can covalently bind to cysteine 909 was from other covalent drugs such as afatinib, osimertinib, and ibrutinib (Figure 2). Afatinib is an irreversible epidermal growth factor receptor (EGFR) inhibitor for wild-type and L858R/T790M double mutations of EGFR [30]. Osimertinib is an EGFR T790M mutant inhibitor [31], and ibrutinib irreversibly binds the protein Bruton’s tyrosine kinase (BTK) [32]. Three drugs in Figure 2 shared a common α,β-unsaturated amide moiety to allow the Michael addition to the target protein via covalent binding. A number of JAK3 covalently bound inhibitors have been reported and some have been studied under various stages of clinical trials [19,20,21,22,23,24,25,26,27,28,29].

From a drug design point of view, it is highly desirable to identify residues that can be utilized for subtype selective binding. It is equally important to evaluate which Covalent Dock method may be more effectively used for the drug design of covalently bound drugs. The answer to the above questions will lay a solid foundation for JAK inhibitor design.

To answer the above questions, in this paper, we used five model proteins to study the subtype selectivity of and the mutational effects on inhibitor binding. We also compared two Covalent Dock methods: one from the Schrödinger software and the other from the MOE software. To evaluate the docking programs and to identify residues that are responsible for selective subtype binding, we used the published JAK3 inhibitors whose activities of JAK1, JAK2, JAK3, and TYK2 were well defined [19].

## 2. Results and Discussion

### 2.1. Multiple Sequence Alignment

The JAK family (JAK1, JAK2, JAK3, and TYK2) has four similar domain structures. To identify the amino acids that may be responsible for the subtype-selective ligand binding, we carried out a multiple sequence alignment of five model proteins: JAK1 (PDB ID: 4E4N) [33], JAK2 (PDB ID: 4D1S) [34], JAK3 C1048S mutant (PDB ID: 5TTV) with covalently bound ligand [35], JAK3 wild-type (PDB ID: 4Z16) with covalently bound ligand [19], and TYK2 (PDB ID: 7UYT) [36] using Clustal Omega (https://www.ebi.ac.uk/Tools/msa/clustalo/ (accessed on 30 June 2022) [37]. The alignment of sequences is given in Figure S1 in the Supplemental Information. Key residues responsible for ligand binding were identified between different JAK subtypes.

Previous studies on developing covalently bound JAK3 inhibitors have revealed that residues Lys855, Leu905, Pro906, Cys909, Asp912, and Arg953 are involved in ligand binding [19,20,21,22,23,26,28]. From the key residues in the multiple sequence alignment (Figure S1, Table 1), one can conclude that the most unique residue for selective JAK3 binding is cysteine 909. In this position, all other three subtypes (JAK1, JAK2, and TYK2) have a serine. The other residues unique to JAK3 are Ser826 and Gln988. However, these two residues were not identified in the ligand binding in our study. Residues that participated in the ligand binding are listed in Table 1. The results suggested that Leu905 can be used to develop subtype-selective ligand binding for JAK3 over TYK2. Residues Arg911 and Asp912 can also be used to design JAK3-selective ligands over JAK1. In these two positions are Lys965 and Glu966 for JAK1.

### 2.2. Molecular Docking

**Figure 3:**
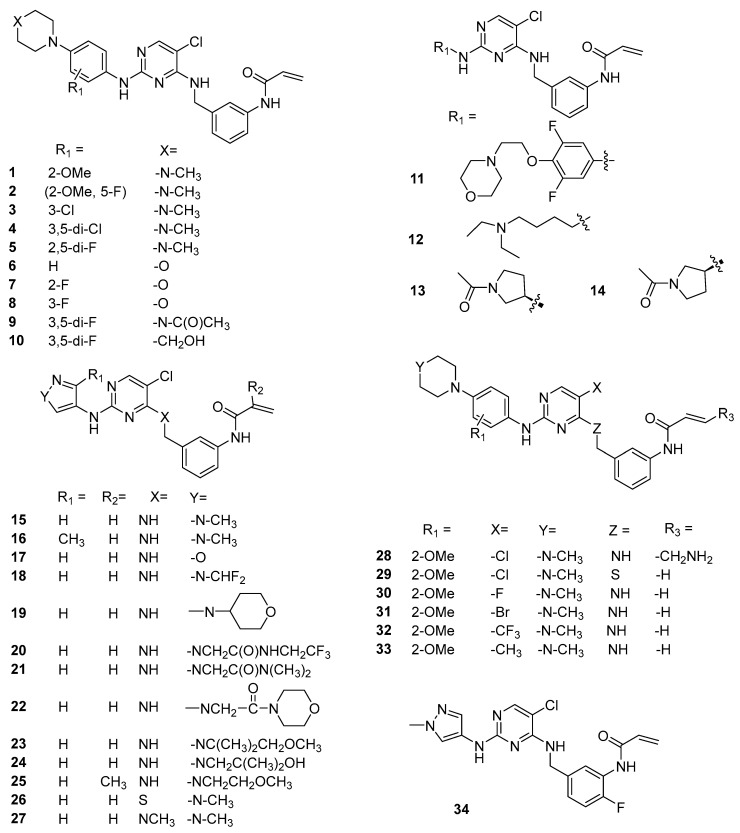
The chemical structures (**1**–**34**) of JAK3 inhibitors taken from Tan’s paper [19].

**Figure 4:**
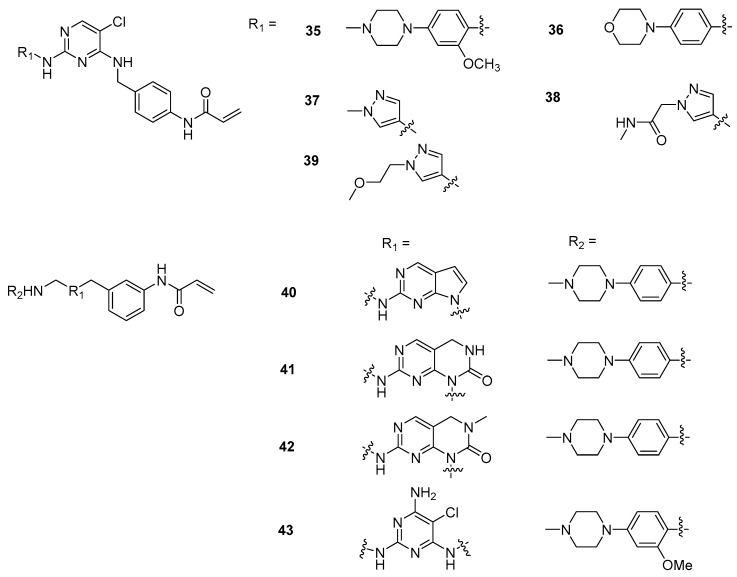
The chemical structures (**35–43**) of JAK3 inhibitors taken from Tan’s paper [19].

We built 43 JAK3 inhibitors using the MOE program [38]. Figure 3 and Figure 4 show that all 43 structures shared a common α,β-unsaturated amide moiety to that observed in the covalent drugs listed in Figure 2. This structural moiety is needed for the covalent binding to the target protein through the Michael addition mechanism.

To identify residues responsible for subtype binding, we docked these 43 ligands against five model proteins representing JAK1 (PDB ID: 4E4N), JAK2 (PDB ID: 4D1S), JAK3 (PDB ID: 5TTV and 4Z16), and TYK2 (PDB ID: 7UYT) using the traditional non-covalent Glide Dock program in the Schrödinger software suite [39]. To determine which Covalent Dock method to use for drug design targeting the residue Cys909 of JAK3, we also used the Covalent Dock protocols in the Schrödinger software suite [39] and in the MOE software [38] to dock the same set of ligand molecules to the 5TTV and 4Z16 model proteins because the JAK3 ligands used in this study were able to form covalent bonds with Cys909 via the Michael addition mechanism. Please note that the reason that we chose the MOE and Schrödinger software for Covalent Dock was based on their availability to our laboratory, though there are some other Covalent Dock programs reported.

#### 2.2.1. Validation of Method

To validate the docking methods we used, we compared the docking-predicted binding affinity (Glide scores) to the experimentally derived free energy of binding (ΔG_exp_), approximated based on the IC_50_s that were reported in the original paper [19]. The free energy of the binding of each ligand was calculated from the experimental IC_50_ (nM) using the following Equation:

ΔG_exp_ (kcal/mol) = RT ln (IC_50_ (nM) × 10^−9^)/1000,

where R = 1.987 cal∙K^−1^∙mol^−1^ and T = 298.15 K.

Among 43 JAK3 inhibitors, Table 2 shows that the mean errors (ΔΔG) between the predicted docking scores and the experimentally derived ΔG_exp_ were very small. The mean error of the 4Z16 Covalent Dock model was 1.21 kcal/mol; that of the 4Z16 non-covalent Glide Dock model was 1.08 kcal/mol. Our calculations showed that both the traditional Glide Dock and the Covalent Dock from the Schrödinger could reliably predict the experimentally derived ΔG_exp_ in the wild-type 4Z16 model.

The mean error of the 4Z16 model between the predicted binding and the ΔG_exp_ from the MOE Covalent Dock program was 1.65 kcal/mol (Table S1, Supplemental Information), which is less than the standard threshold of 2 kcal/mol, suggesting that both the MOE and the Schrödinger Covalent Dock programs were able to reproduce the experimental values and thus should be considered as reliable methods to use for the wild-type 4Z16 model.

#### 2.2.2. Binding Mode of Wild-Type JAK3 Model (4Z16)

**Figure 5:**
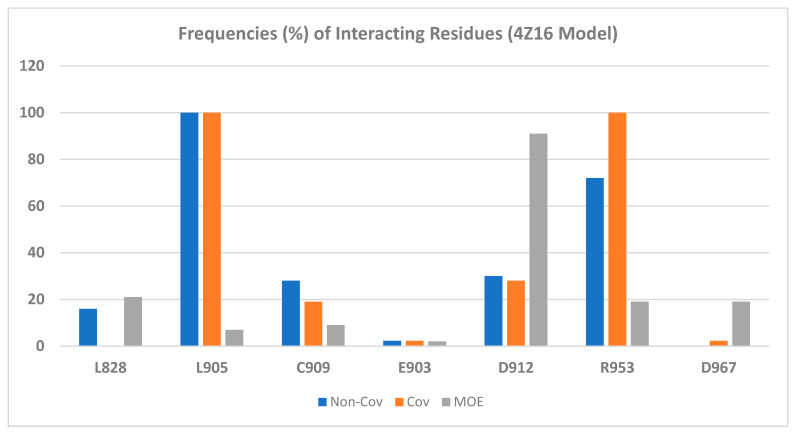
Amino acid frequency (%) of interacting residues of JAK3 inhibitors against the wild-type model 4Z16.

Forty-three JAK3 inhibitors (the structures of the ligands are provided in Figure 3 and Figure 4) were docked to the wild-type JAK3 (4Z16) model using the Glide Dock and the Covalent Dock methods. We also docked these 43 ligands to the JAK3 protein using the MOE Covalent Dock program. After docking was completed, we first analyzed the protein–ligand interactions. Residues interacting with the JAK3 inhibitors are listed in Table 2 for the Schrodinger software results and in Table S1 for the MOE output. We also tabulated the number of residues interacting with the JAK3 4Z16 model, and the frequency of the interacting residues was calculated and is given in Figure 5.

**Table 2:** The glide scores (the NC—non-covalent model) and the Covalent Dock scores (Cov model) (kcal/mol) for the 43 JAK3 inhibitors against the wild-type JAK3 (4Z16) from the Schrödinger software.

Compd.	IC_50_ (nM)	ΔG_Exp_ (kcal/mol)	XP_NC	ΔΔG_NC	Res_NC	XP_Cov	ΔΔG_Cov	Res_Cov
**1**	4.8	−11.35	−9.88	1.47	D912, L905, R953	−10.29	1.06	L905, R953
**2**	46	−10.01	−9.97	0.04	D912, L905, L828	−10.15	−0.14	L905, R953, D912
**3**	2	−11.87	−10.54	1.32	D912, L905, R953	−10.45	1.42	L905, R953, D912
**4**	20	−10.50	−10.96	−0.46	L905, R953	−10.21	0.29	L905, R953, D912
**5**	4.6	−11.37	−11.31	0.06	L905, R953	−10.51	0.86	L905, R953
**6**	1.3	−12.12	−10.64	1.49	L905, R953	−9.74	2.38	L905, R953
**7**	1.4	−12.08	−7.60	4.48	L905, K855	−10.11	1.97	L905, R953
**8**	0.9	−12.34	−9.80	2.54	L905, R953	−10.12	2.22	L905, R953
**9**	3.6	−11.52	−10.25	1.27	L905, C909, L828	−10.42	1.10	L905, R953, R911
**10**	7.4	−11.09	−11.54	−0.45	D912, L905, R953	−10.81	0.29	L905, R953, D912
**11**	6.2	−11.20	−11.38	−0.18	L905, C909, R953	−11.09	0.11	L905, R953, C909
**12**	24	−10.39	−9.52	0.87	L905, L828, R953	−9.25	1.14	L905, R953, P906
**13**	99	−9.55	−10.18	−0.63	L905, R953	−9.67	−0.12	L905, R953
**14**	1600	−7.91	−10.37	−2.46	L905, R953	−10.01	−2.10	L905, R953, C909
**15**	0.6	−12.58	−10.21	2.37	L905, R953	−9.88	2.70	L905, R953
**16**	1.7	−11.96	−10.46	1.50	L905, R953	−10.02	1.94	L905, R953
**17**	2.9	−11.65	−10.18	1.47	L905, R953	−9.68	1.96	L905, R953
**18**	1.4	−12.08	−9.95	2.13	L905, C909, L828	−9.94	2.14	L905, R953
**19**	0.7	−12.49	−9.85	2.64	L905, R953	−9.71	2.78	L905, R953
**20**	0.5	−12.69	−11.60	1.08	L905, C909, R953	−10.89	1.80	L905, R953, C909
**21**	1.1	−12.22	−11.43	0.79	L905, C909, R953	−10.88	1.35	L905, R953, C909
**22**	0.6	−12.58	−10.52	2.06	L905, C909, R953	−10.67	1.91	L905, R953, C909
**23**	0.6	−12.58	−9.62	2.96	L905, L828	−10.71	1.87	L905, R953, C909
**24**	0.6	−12.58	−9.79	2.79	L905, C909, L828	−10.82	1.76	L905, R953, Y904
**25**	7.8	−11.06	−9.50	1.56	L905, C909	−10.69	0.37	L905, R953, Y904
**26**	1.2	−12.17	−10.06	2.11	L905, R953	−9.65	2.52	L905, R953
**27**	0.7	−12.49	−8.88	3.61	L905	−9.71	2.78	L905, R953
**28**	64	−9.81	−10.20	−0.39	L905, D912, R911	−9.27	0.54	L905, R953, D912
**29**	12	−10.80	−10.38	0.43	L905, R953	−9.73	1.07	L905, R953, D912
**30**	30	−10.26	−11.31	−1.04	D912, L905, R953	−10.32	−0.05	L905, R953
**31**	2.3	−11.78	−10.17	1.61	D912, L905, R953	−10.19	1.60	L905, R953, D912
**32**	18	−10.56	−11.05	−0.48	D912, L905, R953	−11.26	−0.69	L905, R953
**33**	31	−10.24	−10.84	−0.59	D912, L905, R953	−10.29	−0.05	L905, R953, D912
**34**	4.4	−11.40	−10.20	1.20	L905, R953	−9.94	1.46	L905, R953
**35**	83	−9.66	−9.28	0.38	D912, L905, R953	−9.88	−0.22	L905, R953, D912
**36**	0.87	−12.36	−9.96	2.40	L905, C909	−9.98	2.38	L905, R953
**37**	0.58	−12.60	−10.25	2.35	L905, R953	−9.33	3.27	L905, R953
**38**	2	−11.87	−11.32	0.55	L905, C909, R953	−10.77	1.10	L905, R953, C909
**39**	1.3	−12.12	−11.33	0.79	L905, C909, R953	−10.23	1.90	L905, R953, C909
**40**	0.7	−12.49	−11.16	1.33	D912, L905, R953	−10.96	1.53	L905, R953, D912
**41**	0.9	−12.34	−11.02	1.32	L905, R953	−10.55	1.79	L905, R953, D912
**42**	7	−11.12	−10.19	0.93	D912, L905	−9.66	1.46	L905, R953
**43**	2720	−7.59	−7.97	−0.38	D912, L905, L828, C909, E903	−9.23	−1.63	L905, R953, D912, E903
Errors				1.08			1.21	
StdEv				1.38			1.19	

**Figure 6:**
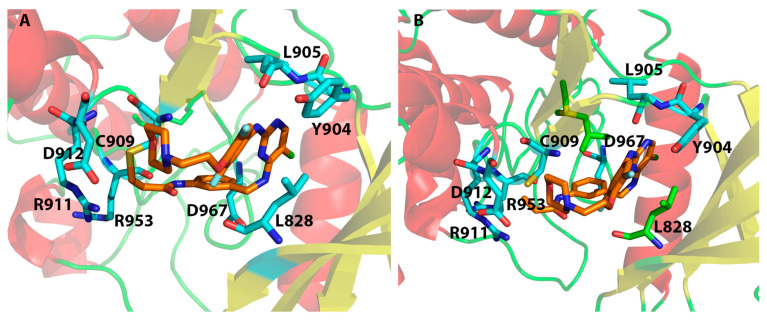
Interactions between JAK3 (4Z16) and compound **11** from the Covalent Dock (**A**) and the regular non-covalent Glide Dock method (**B**). A covalent bond between the β-carbon of the original α,β-unsaturated amide and the Cys909 was found, as shown in (**A**) and highlighted by golden lines.

Both Table 2 and Figure 5 show that residues Leu905 and Arg953 are the most important residues for ligand binding, followed by residues Asp912 and Cys909. Please note that in Figure 5, Cys909 was counted as having non-covalent H-bond interactions with the ligand through hydrogen bond interactions with the main-chain NH of Cys909, though in both the MOE and Schrödinger Covalent Dock methods, a covalent bond was found between each ligand and the sulfur atom of Cys909. Although the important binding residues Leu905, Arg953, Asp912, and Cys909 were identified from both the non-covalent Glide Dock and Covalent Dock methods, the details of ligand binding still differed. The docked poses from the non-covalent Glide Dock were unable to form a covalent bond between Cys909 and the ligand while the Covalent Dock did identify a covalent bond between Cys909 and the β-carbon of the α,β-unsaturated amide via a Michael addition (Figure 6A). There was no surprise that the traditional non-covalent Glide Dock failed to connect the covalent bond with the sulfur atom of Cys909 (Figure 6B). However, it was able to identify the same set of binding residues, Leu905, Arg953, and Cys909 (Table 2, compound **11**). The mean errors were very small: −0.18 and 0.11 kcal/mol for compound **11** against both the Glide Dock and Schrödinger Covalent Dock programs, respectively.

Our docking showed that the presence of an amide, phenol, morpholine, or an ether moiety sometimes would introduce additional interactions with residues such as Asp912, Leu828, and Cys909 (Table 2). The oxygen atom of the morpholine ring of compound **11** was able to form a H-bond with the Cys909 main chain NH group in both the Covalent Dock (Figure 6A) and the Glide Dock (Figure 6B).

**Figure 7:**
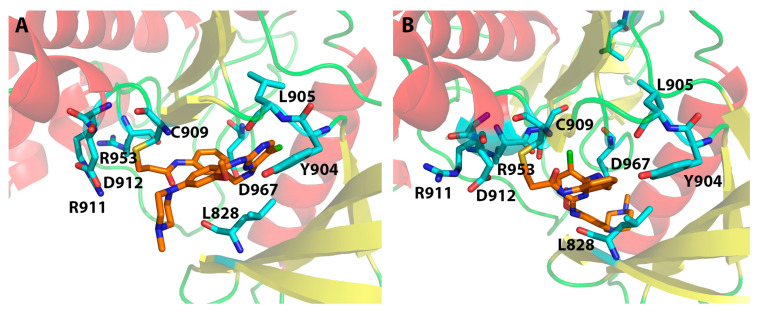
Interactions between JAK3 and compound **1** (4LH in PDB ID: 4Z16) from the Schrödinger Covalent Dock program (**A**) and the MOE Covalent Dock program (**B**). A covalent bond between the β-carbon of the α,β-unsaturated amide and the sulfur atom of the Cys909 is highlighted by the golden lines.

Figure 7 shows that the Covalent Dock programs from both the MOE and Schrödinger software were able to successfully regenerate the covalent bond observed in the crystal structure 4Z16 where the β-carbon in compound **1** (Figure 3) was connected to Cys909. At the same time, the Schrödinger Covalent Dock results identified three H-bond interactions between Arg953, Asp912, and Leu905 with compound **1** (Figure 7A and Table 2). The H-bonds with Leu905 were also present in the X-ray structure of 4Z16 [19]. Our Covalent Dock method identified the new H-bond interactions with Arg953 and Asp912. The errors between the predicted and the experimental value (ΔΔG) for compound **1** and JAK3 were 1.47 kcal/mol for the Schrödinger Covalent Dock method. On the other hand, the predicted error for compound **1** from the MOE Covalent Dock was 2.41 kcal/mol (Table S1, Supplemental Information). Although the overall mean error of the wild-type 4Z16 model was small (1.65 kcal/mol) for the MOE method and each ligand was able to form a covalent bond with Cys909, the prediction of native bound compound **1** (4LH) was higher than predicted with the Schrödinger method.

#### 2.2.3. Binding Mode of the JAK3 C1048S Mutant Model (5TTV)

It is common to observe mutants on proteins, and it is known that mutations cause some structural changes and thus would affect ligand binding. The answer to the question of how a single mutation, in this case, C1048S mutation on JAK3, would affect ligand binding will help guide the selection of a target protein structure for structure-based drug design. To evaluate the mutational effects on ligand binding, we selected a JAK3 C1048S mutant model (5TTV, [35]) in our study. The docking output showed that, in terms of binding affinity, both the traditional Glide Dock and the Schrödinger Covalent Dock programs yielded a much larger error of 2.98 and 2.94 kcal/mol for the 5TTV mutant model, respectively (Table 3). The wild-type 4Z16 model, on the other hand, generated a much smaller error: 1.21 (4Z16, Covalent Dock model) vs. 1.08 kcal/mol (4Z16, non-covalent Glide Dock model, Table 2). Thus, the mutation does have a significant impact on ligand binding in JAK3 based on the Schrödinger method. Therefore, it would be more desirable to select the wild-type protein for JAK3 inhibitor design.

On the other hand, the MOE Covalent Dock method was able to predict the binding of all 43 JAK3 inhibitors with a small mean error of 1.67 kcal/mol for the C1048S mutant model (5TTV). However, we cannot conclude that MOE performed better than the Schrödinger software, because the original paper [19] only reported the wild-type JAK3 activities, and not the activities of the C1048S mutant (5TTV). What we can conclude with confidence is that both the MOE and Schrödinger Covalent Dock programs successfully predicted the binding affinities of ligands against the wild-type JAK3 models (4Z16).

**Figure 8:**
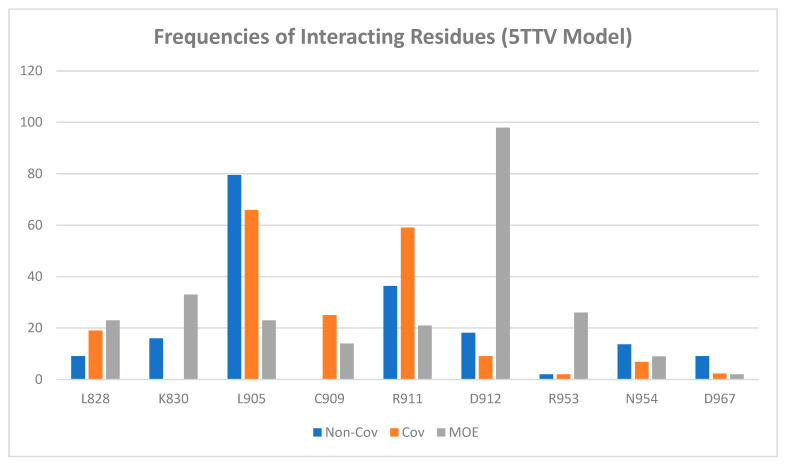
Amino acid frequency (%) of interacting residues of JAK3 inhibitors (5TTV).

The analysis of the protein–ligand interactions between the 5TTV mutant model and the 43 ligands revealed that L905 is still one of the most important residues for ligand binding. This is the same as what was observed in the 4Z16 model. However, Arg953, which was identified as an essential residue for binding in the 4Z16 model, was seldom observed in the 5TTV model; instead, Arg911 was identified as an alternative residue (by the Schrödinger method). The residue Cys909 was still identified as an essential residue for ligand binding by the Covalent Dock method (Figure 8). It needs to point out that MOE identified Asp912 as an important residue interacting with ligands, whether it is in the wild-type 4Z16 (Figure 5) or the mutant 5TTV model (Figure 8). The inspection of the 5TTV/Compound **11** interaction showed that the consistency of identifying Asp912 as an essential interacting residue by the MOE method was due to its proximity to the β-carbon, which was anchored to the Cys909 via a covalent bond (Figure S2, Supplemental Information).

**Figure 9:**
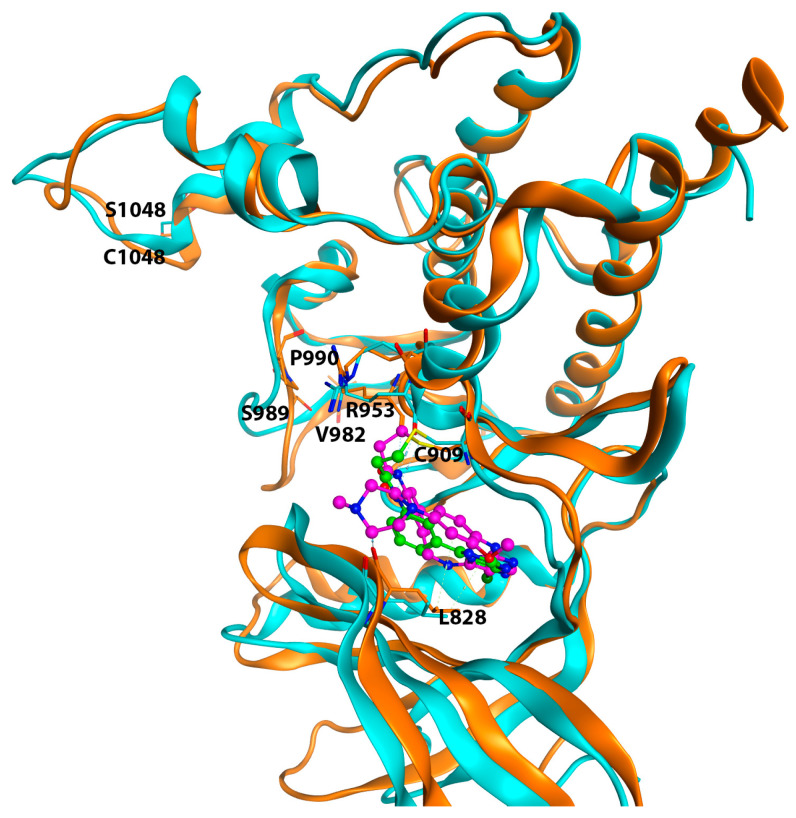
The superposition of the wild-type model 4Z16 (orange) and the C1048S mutant 5TTV (cyan). The bound ligand 4LH of 4Z16 is colored in magenta, and 7KX of 5TTV is colored in green. A covalent bond between the ligand and Cys909 is shown. Residues at 1048, 953, 982, 989, 990, 909, and 828 are labeled.

The superposition of the wild-type 4Z16 and the JAK3 C1048S mutant model 5TTV was carried out in the MOE program, and the root-mean-square deviation (RMSD) for the superposition was 2.0 Å. The α-helices between 4Z16 and 5TTV were aligned very well. However, Figure 9 shows that the mutation at the 1048 position (C1048S) caused a large loop movement in the loop region between residues Val982 and Pro990, which may allow closer interaction between the ligand and the JAK3 4Z16, leading to predicted binding affinities much closer to those of the experimental data than those of the mutant 5TTV model in the Schrödinger models. In addition, the small loop movement of the loop containing Leu828 allowed Leu828 in the 4Z16 model to move closer to the ligand, and thus have stronger hydrophobic interactions. A future study of molecular dynamics simulations would help elucidate the impact of the loop movement caused by the C1048S mutation and at the same time help evaluate whether the residues predicted to be important for ligand binding remain conserved during the simulations.

**Figure 10:**
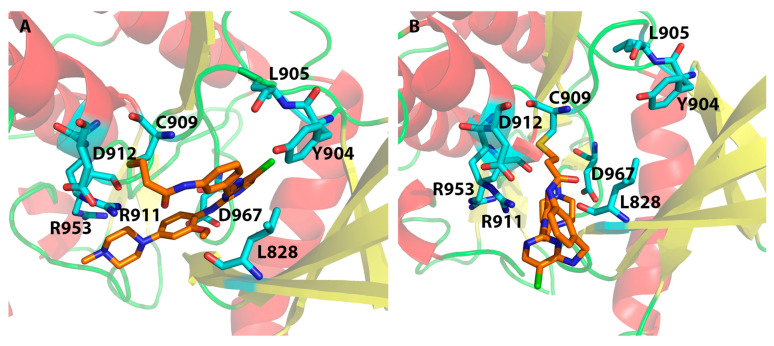
Interactions between JAK3 and compound **1** (in the 5TTV model) under the Schrödinger Covalent Dock program (**A**) and the MOE Covalent Dock program (**B**). A covalent bond between the β-carbon of the original α,β-unsaturated amide and the sulfur atom of the Cys909 is highlighted by golden lines.

The docking study of 5TTV and compound **1** from the Schrödinger Covalent Dock method identified one H-bond interaction between the ligand and Arg911 (Table 3 and Figure 10A). The loss of the H-bond with Leu905 might, in part, contribute to the lower binding affinity with 5TTV (−7.31 kcal/mol vs. −10.29 kcal/mol in the 4Z16 model). Compound **11** interacts with 4Z16 with residues Asp912, Leu905, and Arg953, whereas it binds 5TTV with residue Leu905. The absence of interactions with Asp912 and Arg953 again may, in part, lead to weaker binding of compound **11** with the 5TTV model (−8.70 kcal/mol vs. −11.09 kcal/mol in the 4Z16 model). Similar binding behaviors were also observed in other ligands when they bound to 5TTV, thus contributing to a larger mean error in the 5TTV model. On the other hand, compound **1** was bound to 5TTV (under the MOE Covalent Dock method) with Asp912, Asp967, and Lys830. The smaller mean error (1.25 kcal/mol) for compound **1** may have come from more interactions that were identified (Figure 10B and Table S1).

#### 2.2.4. Binding Selectivity of JAK3 Ligands towards Different Subtypes

To identify which residues are responsible for JAK subtype selective binding, in addition to docking studies on the JAK3 models, we also docked the same set of ligands to model proteins from JAK1 (PDB ID: 4E4N), JAK2 (PDB ID: 4D1S), and TYK2 (PDB ID: 7UYT) using the traditional non-covalent Glide Dock program in the Schrödinger software suite [39]. A Covalent Dock was not performed on these model proteins because these proteins do not contain Cys909; rather, they all contain a serine at the 909th position. The docking scores of the forty-three compounds to the JAK1 (4E4N), JAK2 (4D1S), and TYK2 (7UYT) model proteins are listed in Table S2 in the Supplemental Information. The mean errors of the Glide Dock program for the JAK1 (4E4N), JAK2 (4D1S), and TYK2 (7UYT) model proteins were 1.12, −0.47, and 0.47 kcal/mol (Table S2, Supplemental Information), respectively. The small mean errors in these three JAK subtypes further confirmed the validity of the Glide Dock program.

We also enumerated the interacting residues of the forty-three molecules to the three JAK subtype proteins. By comparing Table 1 and Table 4, it can be easily concluded that Leu905 of JAK3, Leu959 of JAK1, Leu932 of JAK2, and Val981 of TYK2 are important for ligands to bind to all JAK proteins, suggesting that the hydrophobic interaction at this position is important for ligand binding. Other than the unique Cys909, which was able to form a covalent bond with a ligand, other residues of JAK3 that were responsible for ligand binding included Asp912, Arg911, and Arg953. Particularly, residues Arg911 and Asp912 of JAK3 may have selectivity potential over JAK1. At these two positions, JAK1 possessed Lys911 and Glu966, respectively. Table 4 further suggested that Lys911 and Glu966 were not important for JAK1 binding. For JAK1, residues that may contribute to JAK1 selective binding were Phe886, Lys908, Arg1007, Asp1021, Asp1039, and Asp1042. The residues responsible for selective JAK2 binding can be Arg938 and Arg980, and the residues for selective TYK2 binding can be attributed to Arg901, Asp988, and Asp1041 (Table 4). Thus, Asp912 and Arg911 might be used to develop compounds with JAK3 selectivity over JAK1. The selectivity of Asp912 of JAK3 was also confirmed in Hynes et al.’s paper [40].

## 3. Computational Methods

### 3.1. Multiple Sequence Alignment

Protein sequences of JAK1 (PDB ID: 4E4N) [33], JAK2 (4D1S) [34], the JAK3 C1048S mutant (5TTV) [35], JAK3 wild-type (4Z16) [19], and TYK2 (7UYT) [36] were taken from the Protein Data Bank (www.rcsb.org (accessed on 30 June 2022)), and were aligned using the server Clustal Omega (https://www.ebi.ac.uk/Tools/msa/clustalo/ (accessed on 30 June 2022). After alignment, the residues responsible for ligand binding were identified between different JAK subtypes. The multiple sequence alignments are listed in Figure S1 in the Supplemental Information, and the key amino acids among the different JAK subtypes are given in Table 1.

### 3.2. Preparation of Protein Structures

The protein structures of JAK1 (PDB ID: 4E4N) [33], JAK2 (4D1S) [34], the JAK3 C1048S mutant (5TTV) [35], the JAK3 wild-type (4Z16) [19], and TYK2 (7UYT) [36] were downloaded from the RCSB Protein Data Bank (www.rcsb.org, accessed on 25 October 2022) and imported into the MOE program [38]. The missing residues of Glu913-Ser914-Gly915-Gly916-Asn917 and Asp947-Gly948-Gly949-Asn950 in 4E4N were fixed using the loop modeler module in MOE. Similarly, the missing residues of Asn959, Lys1011-Glu1012-Pro1013-Gly1014-Glu1015 in the 4D1S model; the missing residues of Gln858-His859-Ser860; Gly1039-Cys1040-Glu1041-Arg1042 in the 5TTV model; the missing residues of Gly892-Pro893-Gly894-Arg895-Pro896; Glu985-Pro986-Gly987-Ser989; Gly1039-Cys1040-Glu1041-Arg1042-Asp1043-Val1044-Pro1045 in the 4Z16 model; the missing residues of Gly922-Thr923; Ala934-Asp935-Cys936-Gly937-Pro938-Gln939; and Glu1051-Gly1051 in 7UYT were fixed using the loop modeler module in the MOE. All five protein models were then minimized first with the backbone atoms fixed, and then the whole structures were allowed to move and were optimized to reduce the steric hindrance using the Amber14:EHT force field in MOE [41]. The optimized model proteins were then imported to the Maestro program in the Schrödinger software suite [39] for further preparation using the Protein Preparation Wizard in the Schrödinger software to maximize the H-bond interactions of side chains. During this process, residues Glu and Asp were set to a −1 charge, and Arg and Lys were set to a +1 charge, while Asn, Gln, and His were calculated using the Maximize H-bond interaction module to maximize the H-bond network by flipping these residues if necessary, followed by energy minimization using the MacroModel module by using the OPLS3 forcefield. For the model proteins 4Z16 and 5TTV, where the ligands were covalently bound to Cys909, we treated these two proteins in two ways. The first approach was to disconnect the ligand from Cys909, minimize the proteins, and save them as non-covalent models. The second treatment of 4Z16 and 5TTV was to keep the covalent bond between the ligand and Cys909 during minimization and save these two covalent proteins for covalent docking, which will be detailed in the next few paragraphs.

A grid file was generated for each of the five non-covalently bound JAK subtype proteins using the Glide Grid Generation protocol with the bound ligands as centroids of the protein binding pocket.

### 3.3. Preparation of Ligand Structures

Forty-three ligand structures were taken from Tan’s published paper on the JAK3 covalent inhibitors [19], and structures were built using the MOE program [38] with the bound ligand 4LH as a template. Only molecules with IC_50_s activities for JAK1, JAK2, JAK3, and TYK2 were included in this study. In other words, molecules with missing activities on any of the four subtypes and molecules with IC_50_s greater than (>) certain numbers were excluded. Each ligand, after being built, was subject to energy minimization using the force field MMFF94 in the MOE software [38], and then saved to a database. The structures in the database were later imported into the Maestro program in the Schrödinger suite, followed by the treatment with the EPik program [39] to properly protonate or deprotonate based on the calculated pKa, and then were subject to energy minimization using the MacroModel program in the Maestro program [39].

### 3.4. Glide Docking

To study the JAK binding affinity, the 43 inhibitors were docked against the five model proteins (JAK1, 4E4N; JAK2, 4D1S; JAK3 C1048S mutant, 5TTV; JAK3 wild-type, 4Z16; and TYK2, 7UYT) in a non-covalent manner using the traditional Glide Dock program [39]. During the docking process, the scaling factor of the receptor van der Waals for the non-polar atoms was set to 0.8 to allow for the flexibility of the receptor, and Extra Precision was used. All other parameters were used as defaults.

### 3.5. Schrödinger Covalent Docking

To study the effect of the covalent docking, the 43 JAK3 inhibitors were docked to the model proteins 4Z16 and 5TTV using the Covalent Dock program [39] in the Schrödinger software suite. During the setup process, the reaction type was Michael addition, and the scoring function was set to Extra Precision. The top five docked poses were kept in the docking output. Only the docked pose with the best binding affinity was reported. All other parameters were used as defaults.

### 3.6. MOE Covalent Docking

To determine the proper Covalent Dock program to use, in addition to the Schrödinger Covalent Dock program, we also used the MOE Covalent Dock software from the MOE program [38]. Under the MOE DOCK panel, the Covalent module was selected. The refinement was set to Induced Fit and the scoring function to set to London ΔG. The reaction type for the covalent binding was set to the Michael acceptor (1,4-addition) with β-mercapto carbonyl as the product (i.e., forming a covalent bond with sulfur atom of Cys909). The 43 JAK3 inhibitors were docked to the model proteins 4Z16 and 5TTV one at a time. The top five docked poses were kept in the docking output. Only the docked pose with the best binding affinity was reported. All other parameters were used as defaults.

### 3.7. Binding Affinity and Protein-Ligand Interactions

The binding affinity of the protein–ligand complexes was expressed as docking scores. The stronger binders had more negative docking scores. To identify the residues responsible for subtype binding, we tabulated the main interacting residues that provided H-bonds, electrostatic interactions, and/or aromatic interactions. The frequencies of the interacting residues were reported to show which residues have a high frequency in ligand binding. Residues with a high frequency were deemed important for ligand binding.

## 4. Conclusions

Drug resistance in most anticancer treatment is caused by mutations on the target proteins. For instance, the major mechanism for acquired resistance to the first-generation EGFR tyrosine kinase inhibitors such as erlotinib was caused by the T790M mutation on the epidermal growth factor receptor (EGFR) [42]. Afatinib was a covalent EGFR inhibitor that was used to treat metastatic non-small-cell lung cancer (NSCLC) with wild-type and L858R/T790M double mutations of EGFR [30]. Sutanto et al. reported at least 50 covalent drugs that have been approved by the US Food and Drug Administration (FDA) [43].

The advantage of developing covalent drugs is the achievement of isoform- or subtype-selectivity which may be otherwise hard to achieve for most competitive noncovalent inhibitors. JAK3 has been a focus in developing covalent drugs to achieve subtype selectivity and thus to reduce off-target toxicity because of its uniqueness in having a Cys909, which distinguishes it from other subtypes of JAK family members. The cysteine residue has been identified in 27 positions in the active conformation of 211 kinases. Thus, the development of covalent ligands targeting cysteine is of significance in selective inhibitor design [44]. Therefore, identifying a suitable computational program that can accurately predict the binding of covalent ligands will greatly accelerate structure-based drug design via the covalent mechanism.

The molecular docking studies of five models from four JAK subtypes showed that the docking affinity from 4Z16 (JAK3 wild-type model), 4E4N (JAK1), 4D1S (JAK2), and 7UYT (TYK2) agreed well with the experimentally derived binding free energies with small predicted errors. Our findings identified the covalent bond between ligands and Cys909 for all models with the Covalent Dock method. Leu905 of JAK3 and the hydrophobic residue at the same position in different subtypes (Leu959 of JAK1, Leu932 of JAK2, and Val981 of TYK2) is important for ligands to bind to the JAK proteins. Arg911 and Asp912 of JAK3, Asp939 of JAK2, and Asp988 of TYK2 can be used for selective ligand binding over JAK1, which contains Glu966 at the same position. Asp1021, Asp1039, and Asp1042 can be utilized for JAK1-selective ligand design, whereas Arg901 and Val981 may help guide TYK2-selective molecule design. Our docking results of the wild-type 4Z16 model showed that both the Schrödinger and the MOE Covalent Dock programs were able to successfully predict the experimentally derived ΔG, and thus should be able to be used for covalent inhibitor design.

## Figures and Tables

**Table 1:** Residues participating in ligand binding between different subtypes of JAK proteins. The position numbers in the first row refer to the position in JAK3.

Proteins	826	828	833	855	905	909	911	912	953	967	985	988
4E4N (JAK1)	R879	L881	F886	K908	L959	S963	K965	E966	R1007	D1021	D1039	D1042
4D1S (JAK2)	Q853	L855	F860	K882	L932	S936	R938	D939	R980	D994	E1012	E1015
4Z16 (JAK3)	S826	L828	F833	K855	L905	C909	R911	D912	R953	D967	E985	Q988
7UYT (TYK2)	R901	L903	F908	K930	V981	S985	R987	D988	R1027	D1041	E1059	D1062

**Table 3:** The glide scores (the NC—non-covalent model) and the Covalent Dock scores (Cov model) (kcal/mol) for the 43 JAK3 inhibitors against the JAK3 C1048S mutant model (5TTV).

Compd.	IC50 (nM)	ΔGExp (kcal/mol)	XP_NC	ΔΔG_NC	Res_NC	XP_Cov	ΔΔG_Cov	Res_Cov
**1**	4.8	−11.35	−8.90	2.44	L905, D912, R911	−7.31	4.04	R911
**2**	46	−10.01	−8.28	1.73	L905, D912	−7.80	2.21	NA
**3**	2	−11.87	−9.49	2.38	L905, R911, L828	−9.07	2.79	L905, R911, L828
**4**	20	−10.50	−9.59	0.91	L905	−8.99	1.51	L905, D912
**5**	4.6	−11.37	−8.50	2.87	L905	−7.22	4.15	N954
**6**	1.3	−12.12	−6.98	5.15	D967, N954	−8.50	3.62	L905, R911, L828
**7**	1.4	−12.08	−7.52	4.56	D967, N954	−7.42	4.66	R911, R953
**8**	0.9	−12.34	−9.08	3.26	L905	−8.43	3.91	L905, L828
**9**	3.6	−11.52	−6.99	4.52	L905, R911, K830	−8.59	2.93	L905
**10**	7.4	−11.09	−7.66	3.44	Q988, R911, K830	−8.53	2.56	L905, R911, D912
**11**	6.2	−11.20	−10.45	0.74	L905, R916, K830	−8.70	2.50	L905
**12**	24	−10.39	−7.40	2.99	L905, K830	−8.17	2.22	L905, R911
**13**	99	−9.55	−9.59	−0.04	L905, R911, L828	−8.49	1.06	L905, L828, R911
**14**	1600	−7.91	−9.13	−1.22	L905, R911	−7.63	0.27	C909
**15**	0.6	−12.58	−7.34	5.23	L905	−8.36	4.22	L905, R911
**16**	1.7	−11.96	−8.97	2.99	L905, K830	−8.72	3.24	L905, R911
**17**	2.9	−11.65	−8.49	3.15	L905, R911	−8.30	3.35	L905, L828, R911
**18**	1.4	−12.08	−8.93	3.15	L905	−7.46	4.62	L905, C909
**19**	0.7	−12.49	−8.37	4.12	L905, R911	−8.48	4.01	L905
**20**	0.5	−12.69	−9.16	3.52	L905, R911, Y904	−8.38	4.31	L905, R911, C909
**21**	1.1	−12.22	−7.50	4.72	N954, R911, K830	−9.47	2.75	L905, R911, C909
**22**	0.6	−12.58	−8.85	3.73	L905, R911, R916	−9.39	3.19	L905, L828, C909, R911
**23**	0.6	−12.58	−7.61	4.97	L905, R911, K830	−9.70	2.88	L905, C909, R911
**24**	0.6	−12.58	−9.80	2.78	L905, L828	−9.05	3.53	L905, R911
**25**	7.8	−11.06	−9.52	1.54	L905, R953	−9.69	1.37	R911, C909, L905
**26**	1.2	−12.17	−8.75	3.42	L905	−8.28	3.88	L905, R911
**27**	0.7	−12.49	−6.22	6.26	D967	−8.64	3.85	L905, R911
**28**	64	−9.81	−8.41	1.41	L905, N954	−7.08	2.74	N954
**29**	12	−10.80	−6.91	3.90	D912	−8.67	2.14	C909
**30**	30	−10.26	−8.16	2.11	L905, R911, D912	−7.71	2.55	NA
**31**	2.3	−11.78	−8.18	3.60	L905, N954	−8.50	3.28	C909
**32**	18	−10.56	−5.96	4.61	Y904, D967	−7.98	2.58	R911
**33**	31	−10.24	−6.56	3.69	R911	−7.65	2.59	na
**34**	4.4	−11.40	−8.02	3.38	L905	−9.40	2.00	L905, R911
**35**	83	−9.66	−8.80	0.86	L905	−7.81	1.85	R911, D912
**36**	0.87	−12.36	−9.20	3.16	L905	−7.90	4.46	R911, L905
**37**	0.58	−12.60	−9.66	2.94	L905	−7.80	4.80	L828, D967
**38**	2	−11.87	−10.07	1.79	L905, L828	−8.72	3.14	Y904, L905
**39**	1.3	−12.12	−9.87	2.25	L905, R911	−9.67	2.45	L905, C909
**40**	0.7	−12.49	−9.43	3.06	L905, D912	−9.19	3.30	L905, R911
**41**	0.9	−12.34	−6.67	5.67	L905, D912	−8.81	3.53	L905, R911, E903, S989, L828
**42**	7	−11.12	−8.52	2.60	L905, D912	−9.23	1.90	L905, R911
**43**	2720	−7.59	−7.73	−0.14	E903, D912	−7.95	−0.35	D912, R911, E903
Errors				2.98			2.94	
StdEv				1.59			1.15	

**Table 4:** Amino acid frequency (%) of interacting residues of JAK3 inhibitors (5TTV).

JAK1	R879	F886	K908	L959	K965	E966	R1007	D1021	D1039	D1042
frequency (%)	2	30	28	44	0	0	21	35	21	21
JAK2	Q853	F860	K882	L932	R938	D939	R980	D994	E1012	E1015
frequency (%)	0	7	0	67	49	23	42	0	0	0
TYK2	R901	F908	K930	V981	R987	D988	R1027	D1041	E1059	D1062
frequency (%)	53	0	0	67	5	37	5	23	0	0

## Data Availability

Not applicable.

## Supplementary Materials

The following supporting information can be downloaded at https://www.mdpi.com/article/10.3390/ijms24076023/s1.
