# Supplementary material for: Docking and Selectivity Studies of Covalently Bound Janus Kinase 3 Inhibitors
Source: Int J Mol Sci. 2023 Mar 23;24(7):6023. doi: 10.3390/ijms24076023 (PMC10094608; doi:10.3390/ijms24076023)
Supplement: Supplementary file 1 [file ijms-24-06023-s001.zip › ijms-2215848-supplementary.pdf]

Docking and Selectivity Studies of Covalently Bound Janus Kinase 3 Inhibitors

Haizhen A. Zhong [1,*] and Suliman Almahmoud [2,*]

[1] Department of Chemistry, The University of Nebraska at Omaha, 6001 Dodge Street, Omaha, NE 68182, USA
[2] Department of Medicinal Chemistry and Pharmacognosy, College of Pharmacy, Qassim University, Buraidah 51542, Saudi Arabia
* Correspondence: hzhong@unomaha.edu (H.A.Z.); s.a.almahmoud@hotmail.com (S.A.); Tel.: +1-402-554-3145 (H.A.Z.); Fax: +1-402-554-3888 (H.A.Z.)

Figure S1. Multiple sequence alignments of five protein sequences from four different JAK subtypes from the Clustal Omega program.
Table S1. The MOE E_Score1 from the Covalent Dock program (kcal/mol) for the 43 JAK3 inhibitors against the 4Z16 wildtype model, and the JAK3 C1048S mutant model (5TTV).

Figure S2. Interactions between JAK3 and compound **1** (in 5TTV model) from the MOE Covalent Dock program.

Table S2. The glide scores (the NC, non-covalent model) and the Covalent Dock scores (Cov model) (kcal/mol) for the 43 JAK3 inhibitors against the JAK3 C1048S mutant model (5TTV).

```
4D1S_JAK2      --------AFEDRDPTQFEERHLKFLQQLGKGNFGSVEMCRYDPLQDNTGEVVAVKKLQ-  885
4Z16_JAK3      ---------GSCQDPTIFEERHLKYISQLGKGNFGSVELCRYDPLGDNTGALVAVKQLQ-  858
5TTV_JAK3      ----MGHHHHHHQDPTIFEERHLKYISQLGKGNFGSVELCRYDPLGDNTGALVAVKQLQ-  858
4E4N_JAK1      GDIVSEKKPATEVDPTHFEKRFLKRIRDLGEGHFGKVELCRYDPEGDNTGEQVAVKSLKP  912
7UYT_TYK2      --------------PTVFHKRYLKKIRDLGEGHFGKVSLYCYDPTNDGTGEMVAVKALKA  934
                             ** *.:*.** : :**:*:**.*.:  ***  *.**  **** *:
                                        826 828  833                    855

4D1S_JAK2      HSTEEHLRDFEREIEILKSLQHDNIVKYKGVCYSAGRRNLKLIMEYLPYGSLRDYLQKHK  945
4Z16_JAK3      HSGPDQQRDFQREIQILKALHSDFIVKYRGVSYGPGRPELRLVMEYLPSGCLRDFLQRHR  918
5TTV_JAK3      HSGPDQQRDFQREIQILKALHSDFIVKYRGVSYGPGRQSLRLVMEYLPSGCLRDFLQRHR  918
4E4N_JAK1      ESGGNHIADLKKEIEILRNLYHENIVKYKGICTEDGGNGIKLIMEFLPSGSLKEYLPKNK  972
7UYT_TYK2      DCGPQHRSGWKQEIDILRTLYHEHIIKYKGCCEDQGEKSLQLVMEYVPLGSLRDYLPRH-  993
               ..  ::  . ::**:**: *  : *:**:* .   *   ::*:**::* *.*:::* ::
                                                            905 909 911 912

4D1S_JAK2      ERIDHIKLLQYTSQICKGMEYLGTKRYIHRDLATRNILVENENRVKIGDFGLTKVLPQDK  1005
4Z16_JAK3      ARLDASRLLLYSSQICKGMEYLGSRRCVHRDLAARNILVESEAHVKIADFGLAKLLPLDK  978
5TTV_JAK3      ARLDASRLLLYSSQICKGMEYLGSRRCVHRDLAARNILVESEAHVKIADFGLAKLLPLDK  978
4E4N_JAK1      NKINLKQQLKYAVQICKGMDYLGSRQYVHRDLAARNVLVESEHQVKIGDFGLTKAIETDK  1032
7UYT_TYK2      -SIGLAQLLLFAQQICEGMAYLHAQHYIHRDLAARNVLLDNDRLVKIGDFGLAKAVPEGH  1052
                 :.  : * :: ***:** ** ::: :*****:**:*::.:  ***.****:* :  .:
                                                 953           967

4D1S_JAK2      EYYKVKEPGESPIFWYAPESLTESKFSVASDVWSFGVVLYELFTYIEKSKSPPAEFMRMI  1065
4Z16_JAK3      DYYVVREPGQSPIFWYAPESLSDNIFSRQSDVWSFGVVLYELFTYCDKSCSPSAEFLRMM  1038
5TTV_JAK3      DYYVVREPGQSPIFWYAPESLSDNIFSRQSDVWSFGVVLYELFTYCDKSCSPSAEFLRMM  1038
4E4N_JAK1      EYYTVKDDRDSPVFWYAPECLMQSKFYIASDVWSFGVTLHELLTYCDSDSSPMALFLKMI  1092
7UYT_TYK2      EYYRVREDGDSPVFWYAPECLKEYKFYYASDVWSFGVTLYELLTHCDSSQSPPTKFLELI  1112
               :** *::  :**:******.* :  *   ********.*:**:*: :.. ** : *:.::
                    985 988
```

Figure S1. Multiple sequence alignments of five protein sequences from four different JAK subtypes from the Clustal Omega program.

Table S1. The MOE E_Score1 from the Covalent Dock program (kcal/mol) for the 43 JAK3 inhibitors against the 4Z16 wildtype model, and the JAK3 C1048S mutant model (5TTV).

Compd.	**IC50 (nM)**	**ΔG_{Exp} (kcal/mol)**	**4Z16**	**ΔΔG (4Z16)**	**Res_Cov (4Z16)**	**5TTV**	**ΔΔG (5TTV)**	**Res_MOE**
**1**	4.8	-11.35	-8.93	2.41	D912, L956	-10.10	1.25	D912, D967, K830
**2**	46	-10.01	-10.17	-0.16	D912, K830	-9.31	0.70	D912, K830
**3**	2	-11.87	-9.36	2.50	D912, R953,C909	-9.92	1.95	D912, R953, G829
**4**	20	-10.50	-10.84	-0.33	D912, D967	-8.99	1.51	D912, G831, H859
**5**	4.6	-11.37	-9.94	1.43	D912, D967, L828	-11.28	0.10	D912, E903, M902, R911
**6**	1.3	-12.12	-10.93	1.19	L905, L828, G829	-11.57	0.55	D912, C909, L905, Y904, G829, K830
**7**	1.4	-12.08	-9.10	2.98	D912, R953, N954	-9.99	2.09	D912, L828, R953
**8**	0.9	-12.34	-8.96	3.38	K830. G829	-9.92	2.42	D912, R911
**9**	3.6	-11.52	-10.11	1.41	D912, R953	-9.81	1.71	D912, G829, S989
**10**	7.4	-11.09	-8.80	2.29	NA	-8.21	2.89	D912, L956, L828, R953
**11**	6.2	-11.20	-8.83	2.37	D912, L905, L828	-8.96	2.24	D912, D949
**12**	24	-10.39	-10.19	0.20	D912, D967, K855, V836	-9.33	1.07	D912, L828, G829, R953
**13**	99	-9.55	-9.25	0.30	D912	-8.76	0.80	D912
**14**	1600	-7.91	-9.08	-1.17	D912, K855	-9.18	-1.27	D912, R953, K830
**15**	0.6	-12.58	-9.75	2.83	D912, D967	-9.53	3.05	D912, G829
**16**	1.7	-11.96	-9.33	2.64	D912, L970, K855	-8.64	3.32	D912, R911, G831
**17**	2.9	-11.65	-9.23	2.42	D912, D967	-9.19	2.46	D912, GH829
**18**	1.4	-12.08	-9.36	2.71	D967, F833	-9.59	2.49	D912, S989
**19**	0.7	-12.49	-8.36	4.13	D912, R953, G831	-9.99	2.50	D912, L828
**20**	0.5	-12.69	-9.92	2.77	D912, L905, M902, A966	-9.96	2.73	D912, C909, L828
**21**	1.1	-12.22	-9.34	2.88	D912, G829	-8.23	3.99	D912, R953, L828, K830
**22**	0.6	-12.58	-10.75	1.83	D912, L956	-8.42	4.16	D912, K830, F953, N954
**23**	0.6	-12.58	-9.84	2.74	D912, G829	-9.37	3.20	C909, E903
**24**	0.6	-12.58	-8.69	3.89	D912, L956	-9.86	2.71	D912, R953
**25**	7.8	-11.06	-9.48	1.58	D912	-8.33	2.73	D912, G831, S989, R953, G829
**26**	1.2	-12.17	-8.65	3.52	D912, D967, L956	-9.93	2.23	D912, G829, K830, G831
**27**	0.7	-12.49	-9.10	3.39	D912, G831	-10.29	2.20	D912, L828
**28**	64	-9.81	-12.99	-3.18	D912, E871, M902, A966, V836	-9.78	0.03	D912, C909, G829, Q827, D949
**29**	12	-10.80	-9.24	1.57	D912, M902, L828	-9.49	1.32	D912, R953, K830, G829
**30**	30	-10.26	-9.99	0.27	D912, L956, R953	-9.59	0.67	D912
**31**	2.3	-11.78	-10.66	1.12	D912, N954, E903, L828	-9.43	2.35	D912, L828, K830, Q827, R911
**32**	18	-10.56	-10.25	0.31	D912, L828	-9.27	1.29	D912, R911, L828

**33**	31	-10.24	-9.66	0.58	D912	-10.53	-0.29	D912, K830
**34**	4.4	-11.40	-8.29	3.11	D912, R953, K855	-9.43	1.97	D912, K830, C909
**35**	83	-9.66	-11.29	-1.63	D912, N832, D967	-9.93	-0.27	K830, Q988
**36**	0.87	-12.36	-10.42	1.94	D912, V836	-9.95	2.41	D912, R911
**37**	0.58	-12.60	-9.45	3.15	D912, K855	-10.67	1.93	D912, M902, G829
**38**	2	-11.87	-9.22	2.65	D912, K830	-10.55	1.32	D912, R911
**39**	1.3	-12.12	-8.74	3.38	D912, N954	-11.18	0.94	D912, C909
**40**	0.7	-12.49	-10.34	2.15	D912, V836, L828, R953	-11.38	1.11	D912
**41**	0.9	-12.34	-10.31	2.03	D912, L828, R953	-9.39	2.95	D912, K830, R953, S860
**42**	7	-11.12	-10.68	0.44	D912, L828, C909	-10.62	0.51	D912, R911
**43**	2720	-7.59	-10.85	-3.26	D912, L956, G831	-9.64	-2.05	D912, R911, R953, I955
Errors				1.65			1.67	
StdEv				1.73			1.31	

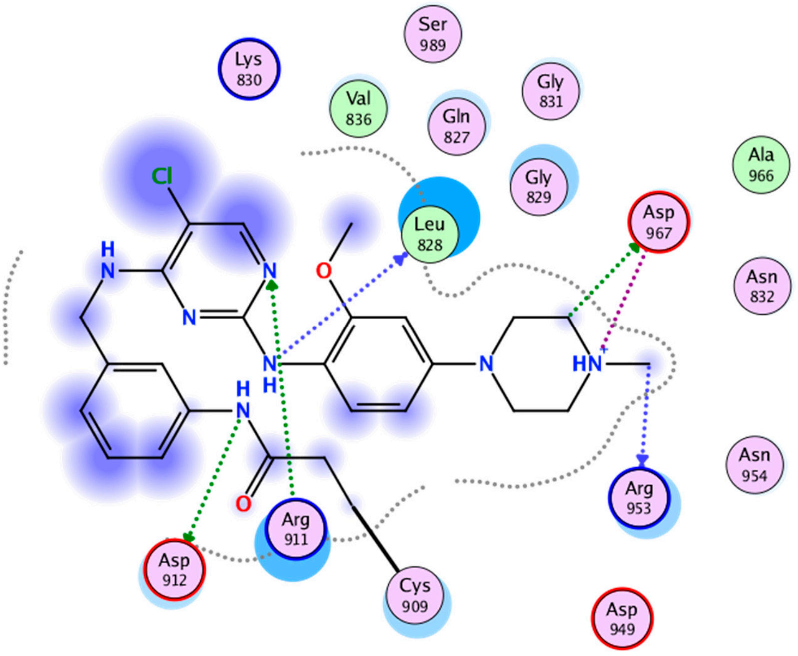

Figure S2. Interactions between JAK3 and compound **1** (in 5TTV model) from the MOE Covalent Dock program.

Table S2. The glide scores (the NC, non-covalent model) and the Covalent Dock scores (Cov model) (kcal/mol) for the 43 JAK3 inhibitors against the JAK3 C1048S mutant model (5TTV).

**Compd.**	**IC_{50} (nM) ΔG_{Exp}**			**ΔG_{Exp} (kcal/mol)**			**Glide 4E4N**	**ΔΔG 4E4N**	**Glide 4D1S**	**ΔΔG 4D1S**	**Glide 7UYT**	**ΔΔG 7UYT**
	JAK1	JAK2	TYK2	JAK1	JAK2	TYK2						
**1**	3124	3194	2266	-7.51	-7.50	-7.70	-6.83	0.68	-6.37	1.13	-6.63	1.07
**2**	3871	6175	3756	-7.38	-7.11	-7.40	-5.77	1.61	-6.16	0.95	-6.50	0.90
**3**	378	692	874	-8.76	-8.40	-8.26	-5.79	2.97	-8.52	-0.12	-6.67	1.60
**4**	1059	2685	992	-8.15	-7.60	-8.19	-6.12	2.03	-10.12	-2.52	-5.67	2.52
**5**	3182	5981	1318	-7.50	-7.13	-8.02	-5.30	2.19	-5.33	1.79	-5.39	2.64
**6**	1857	3704	2353	-7.82	-7.41	-7.68	-7.70	0.12	-9.53	-2.13	-5.87	1.81
**7**	3287	2910	1542	-7.48	-7.55	-7.93	-7.06	0.42	-4.84	2.71	-6.53	1.40
**8**	5195	9087	6613	-7.21	-6.88	-7.07	-8.08	-0.87	-5.97	0.91	-8.93	-1.86
**9**	2004	3290	4666	-7.77	-7.48	-7.27	-7.16	0.61	-6.53	0.95	-9.23	-1.96
**10**	2484	4321	4702	-7.65	-7.32	-7.27	-6.54	1.11	-9.38	-2.06	-6.51	0.76
**11**	2346	2916	4171	-7.68	-7.55	-7.34	-6.36	1.32	-8.02	-0.47	-8.99	-1.65
**12**	17440	30770	17300	-6.49	-6.15	-6.50	-3.11	3.39	-5.14	1.01	-7.95	-1.46
**13**	20330	30780	24780	-6.40	-6.15	-6.28	-7.28	-0.88	-8.22	-2.07	-0.13	6.15
**14**	6792	9695	8170	-7.05	-6.84	-6.94	-7.60	-0.55	-9.36	-2.52	-8.92	-1.98
**15**	2043	3878	1429	-7.76	-7.38	-7.97	-6.33	1.43	-9.78	-2.40	-4.93	3.04
**16**	5198	84897	6502	-7.21	-5.55	-7.08	-7.28	-0.08	-4.40	1.15	-6.13	0.95
**17**	2792	3993	2492	-7.58	-7.36	-7.64	-6.13	1.45	-9.33	-1.97	-6.16	1.48
**18**	1686	3290	994	-7.88	-7.48	-8.19	-6.76	1.12	-7.09	0.39	-8.93	-0.74
**19**	2143	2762	5581	-7.73	-7.58	-7.17	-7.25	0.49	-9.10	-1.52	-7.47	-0.30
**20**	3506	27680	17260	-7.44	-6.22	-6.50	-6.34	1.10	-10.80	-4.59	-8.60	-2.11
**21**	4553	6223	3043	-7.29	-7.10	-7.53	-6.96	0.33	-5.31	1.80	-8.56	-1.03
**22**	8223	19500	4998	-6.94	-6.42	-7.23	-8.66	-1.73	-9.41	-2.99	-7.26	-0.03
**23**	3065	9098	3227	-7.52	-6.88	-7.49	-6.12	1.40	-9.41	-2.53	-8.80	-1.31
**24**	5744	7287	4090	-7.15	-7.01	-7.35	-6.48	0.67	-9.72	-2.71	-7.54	-0.19
**25**	3825	9824	15090	-7.39	-6.83	-6.58	-6.52	0.87	-8.46	-1.63	-8.57	-2.00
**26**	2854	3870	1122	-7.56	-7.38	-8.12	-5.93	1.64	-9.87	-2.49	-7.97	0.15
**27**	3490	4238	3943	-7.44	-7.33	-7.37	-5.95	1.50	-10.16	-2.83	-6.48	0.89
**28**	1823	17240	12680	-7.83	-6.50	-6.68	-6.87	0.96	-7.35	-0.85	-7.59	-0.91
**29**	2324	3101	1318	-7.69	-7.51	-8.02	-6.12	1.57	-6.59	0.93	-6.48	1.55
**30**	11860	11870	5874	-6.72	-6.72	-7.14	-6.23	0.49	-4.60	2.11	-6.63	0.51
**31**	2480	3005	2005	-7.65	-7.53	-7.77	-6.35	1.29	-6.97	0.56	-6.41	1.36
**32**	3640	2426	1936	-7.42	-7.66	-7.79	-6.37	1.05	-5.26	2.40	-2.17	5.62
**33**	3822	4094	3163	-7.39	-7.35	-7.50	-5.40	1.99	-7.86	-0.51	-4.75	2.75
**34**	3624	2326	1598	-7.42	-7.68	-7.91	-6.12	1.30	-9.51	-1.82	-8.75	-0.85
**35**	3974	4996	5452	-7.37	-7.23	-7.18	-6.45	0.92	-6.12	1.11	-7.74	-0.56
**36**	701	874	778	-8.40	-8.26	-8.33	-7.25	1.14	-8.00	0.27	-8.47	-0.13

**37**	2008	4733	754	-7.77	-7.26	-8.35	-5.62	2.16	-5.76	1.50	-7.64	0.72
**38**	7422	10120	6857	-7.00	-6.81	-7.04	-6.09	0.91	-9.11	-2.30	-9.08	-2.04
**39**	3117	5575	3549	-7.51	-7.17	-7.43	-5.42	2.09	-8.05	-0.88	-8.74	-1.31
**40**	1315	2733	4458	-8.02	-7.59	-7.30	-5.15	2.87	-6.60	0.99	-5.16	2.14
**41**	10076	6728	3503	-6.82	-7.06	-7.44	-4.65	2.16	-7.78	-0.73	-6.44	1.00
**42**	25120	8398	5948	-6.27	-6.92	-7.13	-5.14	1.13	-5.18	1.74	-5.73	1.40
**43**	7890	5306	4950	-6.96	-7.20	-7.24	-5.63	1.33	-5.59	1.60	-6.33	0.91
Errors								1.12		-0.47		0.47
StdEv								1.02		1.85		1.93
